# COST OF US EMERGENCY DEPARTMENT AND INPATIENT VISITS FOR FALL INJURIES IN OLDER ADULTS: 2016–2018

**DOI:** 10.1093/geroni/igac059.556

**Published:** 2022-12-20

**Authors:** Lisa Reider, Jason Falvey, Safiyyah Okoye, Joseph Levy

**Affiliations:** Johns Hopkins Bloomberg School of Public Health, Baltimore, Maryland, United States; University of Maryland Baltimore, Baltimore, Maryland, United States; Johns Hopkins School of Nursing, Baltimore, Maryland, United States; Johns Hopkins Bloomberg School of Public Health, Baltimore, Maryland, United States

## Abstract

Falls are a leading cause of injury among older adults. While numerous studies have estimated the economic burden of falls, how health care spending varies by sociodemographic and injury factors is not well understood. The purpose of this study was to describe the average annual frequency of emergency department and inpatient visits and associated costs for fall injuries among older adults in the United States and identify factors associated with higher cost using data from the 2016-2018 National Inpatient Sample and National Emergency Department Sample. The study cohort included encounters with an ICD-10 external cause of injury code for fall (W00-W19). Number of visits was computed using survey weights. Direct visit cost was estimated from charges, applying cost-to-charge and professional fee ratios. On average, an estimated 2.7 million (95%CI: 2.5-2.9) fall-related ED visits and 1.1 million (95%CI: 1.1-1.2) inpatient visits occurred annually. The annual average cost was $1,105 (95%CI: $1,083-$1,127) per ED visit and $18,047 (95%CI: $17,905-$18,189) per inpatient visit totaling $22.9 billion annually. Higher inpatient cost was associated with age (65-74: $20,258 vs 85+: $16,183), gender (men: $19,541 vs. women: $17,181), and race (White: $17,570 vs. Black/Hispanic: $19,602); higher ED cost was associated with age (65-74: $1,009 vs. 85+: $1,198) and dementia diagnosis ($1,369 vs. $1,073). Fifty five percent of inpatient and 25% of ED visits were for fracture which had higher cost compared to sprains, dislocations, and superficial injuries. Results indicate growing number of fall-related admissions and costs underscoring need for targeted prevention and intervention strategies.

